# Preliminary Evaluation of a New Orthotic for Patellofemoral and Multicompartment Knee Osteoarthritis

**DOI:** 10.1155/2021/5923721

**Published:** 2021-09-06

**Authors:** Aleksandra R. Budarick, Emily L. Bishop, Marcia L. Clark, Christopher D. Cowper-Smith

**Affiliations:** ^1^Faculty of Health, Dalhousie University, Halifax, Canada; ^2^McCaig Institute for Bone and Joint Health, Cumming School of Medicine, University of Calgary, Calgary, Canada; ^3^Alberta Hip and Knee Clinic, Alberta Health Services, Calgary, Canada; ^4^Spring Loaded Technology, Halifax, Canada

## Abstract

**Purpose:**

Traditional knee osteoarthritis (OA) braces are usually indicated for a minority of patients with knee OA, as they are only suitable for those with unicompartmental disease affecting the tibiofemoral joint. A new assistive brace design is intended for use in a wider range of knee OA patients with heterogeneous symptoms characteristic of patellofemoral, tibiofemoral, or multicompartmental knee OA. The purpose of this case series was to explore whether the use of this novel “tricompartment offloader” (TCO) brace was associated with clinically relevant improvements in pain and function.

**Materials and Methods:**

A retrospective analysis of individuals with knee OA (*n* = 40) was conducted to assess pain, function, physical activity, and use of medication and other treatments before and after brace use. Validated outcome measures including the Visual Analog Scale (VAS) and Lower Extremity Functional Scale (LEFS) were used to assess pain and physical function (primary outcome measures). Exploratory measures were used to quantify physical activity levels and use of medication and other treatments (secondary outcome measures).

**Results:**

Average total pain (VAS) scores decreased by 36.6 mm and physical function (LEFS) scores increased by 16.0 points following the use of the TCO brace. Overall, 70% of the participants indicated increased weekly physical activity and 60% reported a decrease in their use of at least one other treatment.

**Conclusions:**

Results from this case series suggest that the TCO brace shows strong potential to fill a conservative treatment gap for patients with heterogeneous symptoms of knee OA that are characteristic of patellofemoral or multicompartment disease. Further investigation is warranted.

## 1. Introduction

Osteoarthritis (OA) is the leading cause of disability among older adults [1], and prevalence rates are rapidly increasing. Currently, nearly one in six individuals are affected by OA, and of the approximately 300 joints in the body, the knee is the most commonly affected [2]. Knee OA can affect any of the three compartments within the joint including the medial tibiofemoral (TF), lateral TF, or patellofemoral (PF) compartment. Epidemiological studies suggest that knee OA most commonly affects the PF compartment, either in isolation or in combination with the TF compartments, whereas isolated tibiofemoral OA (TFOA) is relatively less common, accounting for just 5-20% of all knee OA cases [3–5].

The predominant symptom of knee OA is pain, and patients with different compartmental distributions of OA typically exhibit different patterns of pain [5]. High pain levels often lead to functional limitations, decreased physical activity levels, increased use of pain medications, and impaired quality of life [6–8]. As the disease progresses, many individuals opt to undergo total knee replacement (TKR), an invasive and costly procedure which does not guarantee symptom reduction [9–11]. There is no cure for knee OA, so clinical practice guidelines recommend conservative, nonsurgical, and nonpharmacological treatment strategies as the first-line treatment for disease management and pain reduction [12, 13].

Bracing is a common conservative treatment option for pain associated with certain types of knee OA [13]. Most knee OA braces are classified as unicompartment offloaders, which function by redistributing TF joint loading from the affected to unaffected TF compartment during knee extension [14, 15]. Unicompartment offloaders can elicit positive clinical outcomes in patients with unicompartment TFOA who typically experience joint pain that worsens while walking or standing [16, 17]. However, unicompartment offloaders are only clinically indicated for the minority of knee OA patients with isolated unicompartment TFOA [3–5, 18]; these braces are not designed to treat patients who exhibit symptoms of patellofemoral OA (PFOA), who typically experience pain that gets worse during weight-bearing flexion [19–21]. Additionally, while patellar realignment sleeves have been tested with mixed results as a solution for PFOA, they are not indicated unless the patella is misaligned, and they do not address multicompartment disease [22, 23]. As a result, there is a treatment gap in the available knee bracing options for the majority of patients who exhibit symptoms of PFOA or multicompartment disease, including combined PFOA and TFOA, bicompartment TFOA, or tricompartment knee OA [3–5].

**Figure 1:**
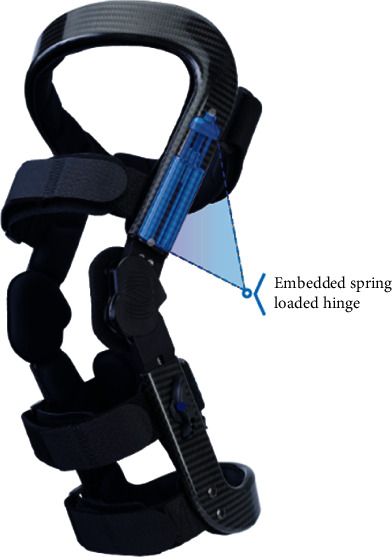
Image of the tricompartment offloader (TCO) knee brace with spring-loaded hinge.

To address this gap, an assistive “tricompartment offloader” (TCO) brace was recently developed to address knee pain and function in patients with knee OA, independent of compartmental distribution (Figure 1). The brace is designed to simultaneously reduce PF and TF joint contact forces during knee-flexed weight-bearing activity when joint forces are known to be highest [24]. The TCO brace functions by storing potential energy in springs during weight-bearing knee flexion and applying an external moment to the back of the leg to assist knee extension. The result is a brace capable of providing a clinically meaningful [25] unloading effect in both the TF and PF compartments of the knee during flexion, equivalent to what would be achieved by a 45 lb body weight reduction [24, 26].

The purpose of this case series was to explore whether the use of the TCO brace was associated with clinical benefits in individuals with heterogeneous symptoms of knee OA for the first time. Unlike prior bracing studies, this study included individuals with heterogeneous symptoms characteristic of TFOA, PFOA, or combined TFOA and PFOA [27, 28]. The primary outcomes were patient-reported knee pain and physical function scores. Secondary outcomes included physical activity levels, use of medication, and frequency of other therapies. We hypothesised that all brace users (independent of their pattern of knee OA) would report significant reductions in pain [29, 30] and improvements in function [31] following use of the TCO brace.

## 2. Materials and Methods

Individuals (*n* = 55) were randomly selected to participate in the survey from a dataset of patients who had been prescribed a brace in the preceding 9 months (*n* = 436). Participants were included if they confirmed a medical diagnosis of knee OA and if they owned and used the TCO knee brace (Levitation® Tri-Compartment Offloader™, Spring Loaded Technology Inc., Halifax, NS) for a minimum of 1 month at the time of survey. Participants were excluded if they were not using the brace at the time of the survey (e.g., due to a subsequent unrelated injury or surgery), if they did not use the brace personally (e.g., they purchased the brace on behalf of someone else), or if they did not confirm a medical diagnosis of knee OA. Participants were asked to read and sign an informed consent form prior to participation. Each participant was assigned a unique identification code, and all responses were deidentified prior to data processing and analysis. The survey results maintain the confidentiality of users, and the study was conducted according to the principles of the Declaration of Helsinki (World Medical Association, revised in 2013).

### 2.1. Data Collection

An online survey (Appendix 1) was administered using Qualtrics XM software. Demographic information including age, height, and body mass was collected. Brace wear characteristics were collected to determine the length of time users had been wearing the brace, as well as average days per week and hours per wear. The primary research interest was to determine whether the use of the TCO brace was associated with improvements in pain and function in a group of individuals with heterogeneous symptoms of knee OA.

To explore whether the brace effects were dependent on participants' different patterns of pain, participants selected the most appropriate description of the location of their knee pain based on clearly differentiated descriptions of PFOA and TFOA symptoms [27, 28]. Patient-reported knee pain location has been associated with symptoms and functional limitation [28] as well as structural imaging measures [32] in knee OA populations. Based on the reported location(s) of their knee pain, participants in this study were separated into three groups for exploratory analysis: PFOA, TFOA, or combined TFOA and PFOA [27, 28]. Participants were also asked to select the severity of their knee pain based on descriptions of mild, moderate, and severe symptoms [33] and reported whether they had used a brace for one (unilateral) or both (bilateral) knees.

Participants were asked to provide responses for the period before they started using the brace and in their current state (i.e., after using the brace). Self-reported knee pain was evaluated using the standardised 100 mm Visual Analog Scale (VAS). The VAS is a valid and reliable standard for assessing knee pain scores [34] and was used to assess pain for activities of interest, as described elsewhere [35]. Pain during activities of daily living was assessed using benchmarks (i.e., while sitting and worst pain experienced in the last week) and during tasks that typically aggravate PFOA (going up or down stairs, standing from seated, crouching or squatting, and hiking or walking on uneven terrain) and TFOA (walking long distances on a flat surface) [27]. Scores ranged from 0 mm “no pain” to 100 mm “worst pain imaginable.” Participant's total pain score at each time point was the sum of pain scores from all activities, excluding benchmark values, divided by the number of activities for which they completed a pain score rating.

Functional status scores were evaluated using the Lower Extremity Functional Scale (LEFS), a 20-item questionnaire applicable to a wide range of individuals with orthopaedic conditions of the lower extremity [31]. The LEFS is reliable and valid for individuals with knee OA [36] and has been used to evaluate knee extension assist (KEA) braces designed for end-stage knee OA [37]. Each item is scored on a 5-point scale from 0 “extreme difficulty/unable to perform activity” to 4 “no difficulty.” An 18-item questionnaire was used for this study, which has been deemed sufficient for estimating functional status in individuals with lower extremity impairments [38]. Total LEFS scores (sum of all items) range from 0 to 76 points, with higher scores representing higher levels of function.

Secondary exploratory questions were developed to assess physical activity levels, use of medications, and use of other therapies. Participants were asked to report their estimated physical activity levels in hours per week. For medication use, participants were asked to list all prescription, over the counter, or other drugs used to reduce knee pain or inflammation. Total weekly medication frequency was calculated as the sum of all instances of medications used per week. Participant responses were pooled into the following categories: 0-7, 8-14, 15-21, 22-28, and 29-35 instances/week. Other therapies were recorded using checkboxes to identify which modalities participants used or considered using before TCO brace use and which therapies they had reduced, delayed, or eliminated after TCO brace use. Categories included injections, allied health services, minor surgery, major surgery, recreational drugs, or other aids (Appendix 1).

### 2.2. Statistical Analysis

Data were presented as group means and standard deviations for continuous variables and medians and interquartile ranges for categorical variables. An alpha of 0.05 was used for all tests, and a Bonferroni correction was used to adjust for multiple comparisons. Participants were divided into three groups (TFOA, PFOA, or combined TFOA and PFOA) to explore possible differences between symptom groups.

A one-way ANOVA was conducted to assess differences in participant demographics between groups. Normality of continuous variables was tested using *Q*-*Q* plots. Total pain and LEFS scores were normally distributed. A log transformation was applied to physical activity level to reduce skewness, resulting in a normal distribution. All statistical analyses for physical activity level were performed on the log-transformed data. Paired *t*-tests were used to assess differences over time (before and after brace use) in total pain score, LEFS score, and physical activity levels for all participants. A chi-square test was used to assess differences over time in weekly medication frequency.

Between-group differences were assessed while controlling for potential covariates. A two-way ANCOVA was used to assess differences between groups and duration of brace use and their interaction on the change in total pain score and LEFS score while controlling for baseline activity level. Descriptive statistics were compiled and presented by group to identify trends in frequency of medication and other therapies.

## 3. Results

Of the 55 individuals invited to participate, 10 individuals were excluded (8 did not confirm a medical diagnosis of knee OA, 1 stopped using the brace, and 1 did not use the brace personally), leaving 45 eligible individuals. Forty participants completed the survey, resulting in a response rate of 88.9% (Table 1). Reported symptoms were consistent with TFOA in 15% of the participants (*n* = 6), PFOA in 22.5% of the participants (*n* = 9), and combined TFOA with PFOA in 62.5% of the participants (*n* = 25), which is consistent with the ratios observed in epidemiological knee OA data [3, 4]. For severity, 20% of the participants (*n* = 8) reported mild, 35% (*n* = 14) reported moderate, and 45% (*n* = 18) reported severe knee OA symptoms. Most participants (75%, *n* = 30) used a TCO brace on one knee (unilateral), while the remainder (25%, *n* = 10) used TCO braces for both knees (bilateral). Median categorical brace wear characteristics showed that participants had used the brace for 3-5 months (range: <1 month to 6+ months), approximately 4 days per week (range: 1 to 7 days) for a duration of 3 hours per day (range: <1 hour to all day). There were no significant differences in age, height, body mass, or body mass index (BMI) between groups (Table 1). Results are reported for the overall group, as well as each symptom group (TFOA, PFOA, and combined) to enable interpretation of between-group trends.

### 3.1. Pain Scores

**Figure 2:**
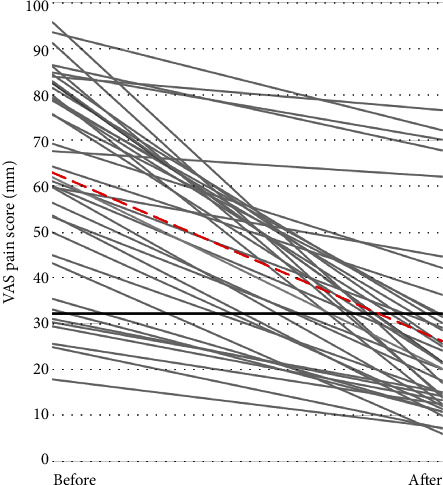
Changes in Visual Analog Scale (VAS) pain score (mm) over time (before and after TCO brace use). Each grey line represents one participant, and the red dashed line represents the overall group average. The black line represents the patient acceptable symptom state (PASS) threshold of 32.3 mm [29]. Values below this threshold indicate clinical significance, where patients consider themselves well.

Average total VAS scores decreased from 62.5 ± 23.3 mm before brace use to 25.9 ± 20.2 mm following brace use (*p* < 0.001). All 40 participants indicated a decrease in pain, with reductions ranging from 5.6 mm to 78.6 mm (Figure 2). There were no significant differences between symptom groups (*p* = 0.109), duration of brace use (*p* = 0.346), or their interaction (*p* = 0.332) on change in total VAS scores when controlling for baseline activity level. Within the symptom-defined groups, total VAS scores decreased by 20.5 mm, 31.6 mm, and 40.7 mm for the TFOA, PFOA, and combined groups, respectively (Table 2). Group trends for each activity are shown in Supplemental Figure 1.

### 3.2. Lower Extremity Function

**Figure 3:**
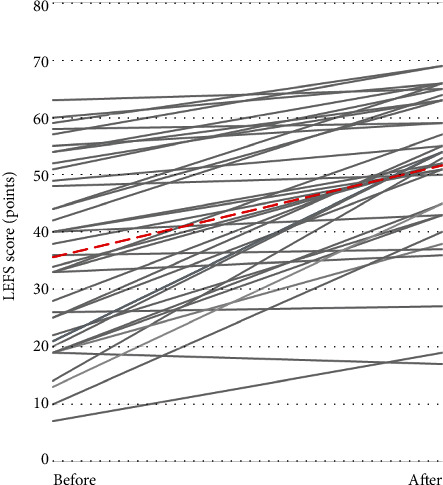
Changes in Lower Extremity Functional Scale (LEFS) scores (points) over time (before and after TCO brace use). Each grey line represents one participant, and the red dashed line represents the overall group average.

Average LEFS scores increased from 35.6 ± 15.8 points before brace use to 51.6 ± 12.7 points following brace use (*p* < 0.001). Thirty-nine of the forty participants (97.5%) showed increased LEFS scores, with changes ranging from −2 points to +39 points (Figure 3). There were no significant differences between symptom groups (*p* = 0.496), duration of brace use (*p* = 0.661), or their interaction (*p* = 0.290) on change in LEFS scores when controlling for baseline activity level. LEFS scores increased by 11.5 points, 15.3 points, and 17.3 points for the TFOA, PFOA, and combined symptom groups, respectively (Table 2).

### 3.3. Physical Activity Levels

Weekly physical activity levels increased from 15.7 ± 15.0 (log transformed: 1.03 ± 0.37) weekly hours before brace use to 23.5 ± 19.9 (log transformed: 1.23 ± 0.36) weekly hours following brace use (*p* < 0.001). Twenty-eight of the forty participants (70%) indicated increased duration of physical activity per week, with changes ranging from −2 hours to +40 hours. Weekly physical activity levels increased by 2.2 hours, 7.3 hours, and 9.4 hours for the TFOA, PFOA, and combined groups, respectively (Table 2).

### 3.4. Medication Use

Medication use was reported by 65% of the participants (*n* = 26) at baseline, and weekly medication frequency was significantly reduced following brace use in these participants (*p* < 0.001). The most common types of medication reported at baseline included ibuprofen (30%, *n* = 12), acetaminophen (20%, *n* = 8), and other NSAIDS (e.g., naproxen and meloxicam; 15%, *n* = 6). In total, 27% (*n* = 7) of the participants who reported using medications prior to brace use decreased their medication frequency by at least one categorical level after brace use, and no participants reported an increase in medication use (Table 3).

### 3.5. Use of Other Therapies

Use of other therapies was reduced following TCO brace use. Overall, 60% of the participants reported that they had reduced, delayed, or eliminated at least one form of other treatment after using the TCO brace (Table 4). The most frequent groups of therapies used or considered before brace use were allied health services (73%), injections (43%), and aids (30%). The therapies most frequently identified as being reduced following TCO brace use were allied health services (35%), injections (20%), and major surgery (13%). Of the 10 participants who indicated they were considering TKR before the brace, 50% indicated that TCO brace use had reduced, delayed, or eliminated their need for TKR.

## 4. Discussion

The TCO is the first offloader brace designed for patients with PFOA or multicompartment disease [24], and the potential clinical benefits of the TCO have not been previously evaluated. In the current study, participants experienced statistically significant and clinically relevant improvements in pain and function following TCO brace use, independent of the pattern of knee OA exhibited. Users also reported increased physical activity and decreased use of medications and other therapies. These encouraging results suggest that TCO may help fill a conservative treatment gap for patients with PFOA or multicompartment disease [39]. The following discussion provides interpretation of the study results and highlights limitations associated with this first clinical study on the TCO brace.

### 4.1. Pain Scores

Participants reported statistically significant reductions in knee pain following TCO brace use. As described elsewhere, the patient acceptable symptom state (PASS) is the value below which affected individuals typically consider themselves well [29]. For knee-specific VAS pain scores, the PASS is 32.3 mm [29] and the minimal clinically important improvement (MCII) is 19.9 mm on a 100 mm VAS [29, 30]. The TCO brace positively influenced pain scores to decrease overall scores below the PASS threshold (average = 25.9 mm). Additionally, the changes observed in overall pain scores were greater than the MCII (average = 36.6 mm), indicating a clinically meaningful difference in symptoms.

The magnitude of change in pain scores for the TFOA symptom group was similar to that noted for a unicompartment offloader brace with KEA functionality [37]. The TFOA group in this study experienced a decrease in total pain of 20.5 mm, and use of a unicompartment offloader/KEA brace in another study decreased pain scores by 19.0 mm [37]. Notably, although the PFOA and combined symptom groups in this study reported higher baseline VAS scores, pain was nonetheless reduced to a level below the PASS threshold (Table 2). The greatest decreases in pain were therefore reported by the PFOA and combined symptom groups, who experienced decreases of 35.8 and 40.7 mm, respectively. Knee pain is a hallmark symptom of knee OA and greatly impacts function and quality of life, so decreasing pain in these individuals is a primary treatment objective for overall wellbeing [12, 40].

### 4.2. Lower Extremity Function

Use of the TCO brace provided statistically significant improvements in participant-reported physical function, eliciting an increase of 16 points in LEFS scores. This change surpasses the minimal clinically important difference (MCID) of 9 points [31], indicating that use of the TCO brace provides a significant and clinically relevant improvement in physical function for individuals with heterogeneous symptoms of knee OA. The TFOA symptom group in this study recorded an average baseline LEFS score of 45.5 points with an increase of 11.5 points after TCO brace use. In the PFOA and combined symptom groups, greater absolute improvements were observed, with increases in LEFS scores of 15.3 and 17.3 points, respectively, although between-group differences were not statistically significant. Lower extremity function is necessary to maintain normal activities of daily living, so improving physical function is critical for the functional independence of individuals with knee OA.

### 4.3. Physical Activity Levels

Participants reported increased physical activity levels following TCO brace use. Obtaining adequate physical activity remains a challenge for most individuals with knee OA, and the majority do not meet physical activity guidelines [7, 41]. Physical activity has been shown to improve physical functioning and reduce pain and disability in individuals with knee OA [42]. Physical activity interventions can also lead to weight loss and strength improvements, both of which improve knee OA outcomes, including those related to joint loading, pain, function, and mobility [43]. Therefore, the TCO brace may provide an effective tool to enable adults with activity-related knee pain to participate in physical activity and/or physical rehabilitation programs.

### 4.4. Medication Use

Use of the TCO brace significantly decreased the reported weekly frequency of medication use. Knee OA treatment guidelines suggest the combination of pharmacological and nonpharmacological therapies, but caution the long-term use of certain common pharmacological therapies [13]. Additionally, the use of medication for OA represents a significant health care expense, totaling US $15.6 billion in the USA [44] and is projected to reach up to CA $1.6 billion by 2031 in Canada [45]. For these reasons, the use of nonpharmacological therapies remains the first-line treatment for knee OA [13]. This study suggests that the TCO brace may allow some individuals with knee OA symptoms to reduce their use of pain medications, resulting in a reduced risk of long-term side effects and potential for significant cost savings.

### 4.5. Use of Other Therapies

Use of the TCO brace decreased reported use of other therapies across all categories. The largest reported reductions were observed in allied health services, injections, and major surgery. Allied health services provide some of the most common rehabilitation therapies for knee OA including physiotherapy and chiropractic therapy. However, the costs associated with accessing these services are high, with estimated out-of-pocket costs reaching CA $1.2 billion and rehabilitation costs reaching CA $0.7 billion by 2031 [45]. Given the unloading and assistive capability of the TCO brace [24], it may be possible to use the TCO to complement the benefits of other rehabilitative therapies, including permitting full range of motion while weight-bearing, increasing quadriceps strength, and promoting soft-tissue repair [46].

Use of the TCO brace also appears to have influenced the number of individuals considering surgery as a treatment option. Since TKR is invasive and does not guarantee symptom relief for all individuals [11], treatment guidelines recommend TKR only after nonpharmacological and pharmacological therapies fail to provide adequate pain relief or functional improvements [13]. Results from this study demonstrate that some individuals with knee OA symptoms were no longer considering surgery after using the TCO brace; however, future studies with larger sample sizes are required to determine the possible extent of this effect.

### 4.6. Clinical Relevance

Use of the TCO brace resulted in clinically relevant improvements in pain and function [47]. Importantly, these benefits were achieved in patients with heterogeneous symptoms of knee OA, independent of whether their symptoms were characteristic of TFOA, PFOA, or combined TFOA and PFOA. Taken together, the results indicate the TCO brace shows promise to fill a conservative treatment gap for patients with PFOA and multicompartment knee OA (i.e., individuals who are unlikely to benefit from unicompartment offloaders or PF realignment sleeves [18, 22, 23, 39]). The outcome of this case series provides encouraging evidence to support conducting larger scale, controlled trials. Future research should also examine the potential value of the knee extension assistance and pain benefits offered by the TCO brace (e.g., to overcome arthrogenic muscle inhibition during physical rehabilitation from knee OA and other musculoskeletal injuries).

### 4.7. Limitations

Several limitations of this case series should be noted. Unicompartment offloader braces are not designed to unload the PF joint and may exacerbate symptoms of patients with bicompartment TFOA [48]; consequently, a unicompartment offloader control group was not included. However, future research could compare TCO outcomes to PF realignment braces, compressive knee sleeves, or a standard of care (e.g., a physiotherapy regimen). Additionally, the retrospective study design relied on participants' memory for scores before TCO brace use, which may have resulted in recall bias [49]. Finally, participants were grouped based on patient-reported symptom locations for exploratory analysis [28, 32], but future studies could include imaging (e.g., radiographs, ultrasound) to identify the affected knee compartment(s). Overall, this case series provides a preliminary understanding of the potential benefits of the TCO brace for a group of individuals with heterogeneous symptoms of knee OA and represents an important first step to help warrant future trials.

## 5. Conclusions

In a group of individuals exhibiting heterogeneous symptoms of knee OA, pain scores, medication use, and reliance on other therapies decreased following TCO brace use, while physical function and physical activity levels increased. Future research is warranted to further examine the clinical value of the assistive TCO brace.

## Figures and Tables

**Table 1:** Participant demographic information by symptom group. *p* values reported for the main effect of symptom group (*α* = 0.05).

Variable	TFOA (*n* = 6)	PFOA (*n* = 9)	Combined (*n* = 25)	Overall (*n* = 40)	Sig. group (*p* value)
	Mean (SD)				
Age (years)	54.3 (11.7)	60.1 (8.6)	60.3 (10.5)	59.4 (10.2)	*p* = 0.439
Height (m)	1.73 (0.11)	1.73 (0.08)	1.74 (0.09)	1.74 (0.09)	*p* = 0.924
Body mass (kg)	78.8 (16.0)	84.4 (12.3)	94.2 (20.7)	89.7 (19.1)	*p* = 0.132
BMI (kg/m^2^)	26.1 (3.7)	28.1 (4.3)	30.7 (4.8)	29.4 (4.8)	*p* = 0.071

**Table 2:** Pain (VAS) scores, LEFS scores, and physical activity levels before and after brace use. *p* values reported for the main effect of time (before vs. after TCO brace use). Statistical significance indicated with ∗ (*α* = 0.05).

Measure	Time point	TFOA (*n* = 6)	PFOA (*n* = 9)	Combined (*n* = 25)	Overall (*n* = 40)	Sig. time (*p* value)
		Mean (SD)				
Total VAS scores (mm)	Before	45.0 (27.1)	60.1 (23.6)	67.5 (21.0)	62.5 (23.3)	*p* < 0.001^∗^
	After	24.5 (22.9)	24.3 (15.0)	26.7 (21.7)	25.8 (20.2)	
LEFS scores (points)	Before	45.5 (16.0)	37.3 (16.4)	32.6 (15.2)	35.6 (15.8)	*p* < 0.001^∗^
	After	57.0 (10.6)	52.7 (10.3)	50.0 (13.8)	51.6 (12.7)	
Physical activity levels (weekly hours, raw score)	Before	17.7 (26.0)	15.6 (15.0)	15.2 (12.2)	15.7 (15.0)	N/A
	After	19.8 (4.8)	22.9 (18.2)	24.6 (20.0)	23.5 (19.9)	
Physical activity levels (weekly hours, log transform)	Before	0.97 (0.48)	1.00 (0.43)	1.06 (0.33)	1.03 (0.37)	*p* < 0.001^∗^
	After	1.11 (0.39)	1.23 (0.38)	1.26 (0.35)	1.23 (0.36)	

**Table 3:** Weekly medication frequency before and after brace use.

Group		Time point	Medication frequency groups				
			1 (0-7 instances per week)	2 (8-14 instances per week)	3 (15-21 instances per week)	4 (22-28 instances per week)	5 (29-35 instances per week)
			Percentage of group				
TFOA	*n* = 6	Before	100%	0%	0%	0%	0%
		After	100%	0%	0%	0%	0%
PFOA	*n* = 9	Before	67%	11%	11%	0%	11%
		After	89%	0%	0%	0%	11%
Combined	*n* = 25	Before	56%	20%	16%	0%	8%
		After	68%	20%	12%	0%	0%
Overall	*n* = 40	Before	65%	15%	13%	0%	8%
		After	78%	13%	8%	0%	3%

**Table 4:** Use of other treatments before and after TCO brace use.

Group		Before TCO brace use						Reduced following TCO brace use					
		Injections	Allied health services	Minor surgery	Major surgery	Drugs	Aids	Injections	Allied health services	Minor surgery	Major surgery	Drugs	Aids
		Percentage of group											
TFOA	*n* = 6	50%	67%	0%	0%	0%	50%	17%	50%	0%	0%	0%	17%
PFOA	*n* = 9	33%	89%	0%	11%	22%	44%	22%	33%	0%	11%	11%	11%
Combined	*n* = 25	44%	68%	20%	36%	16%	20%	20%	32%	12%	16%	8%	4%
Overall	*n* = 40	43%	73%	13%	25%	15%	30%	20%	35%	8%	13%	8%	8%

## Data Availability

The data that support the findings of this study are available from the corresponding author upon reasonable request.

## Supplementary Materials

Supplementary 1 Supplemental Figure 1: change in VAS pain scores (average + SD) from before to after using the TCO brace for specified activities of daily living by symptom group.

Supplementary 2 Appendix 1: retrospective survey.
