# Supplementary material for: Preliminary Evaluation of a New Orthotic for Patellofemoral and Multicompartment Knee Osteoarthritis
Source: Rehabil Res Pract. 2021 Sep 6;2021:5923721. doi: 10.1155/2021/5923721 (PMC8441257; doi:10.1155/2021/5923721)
Supplement: Supplementary 1 — Supplemental Figure 1: change in VAS pain scores (average + SD) from before to after using the TCO brace for specified activities of daily living by symptom group. [file 5923721.f1.docx]

**Supplemental Figure 1.**


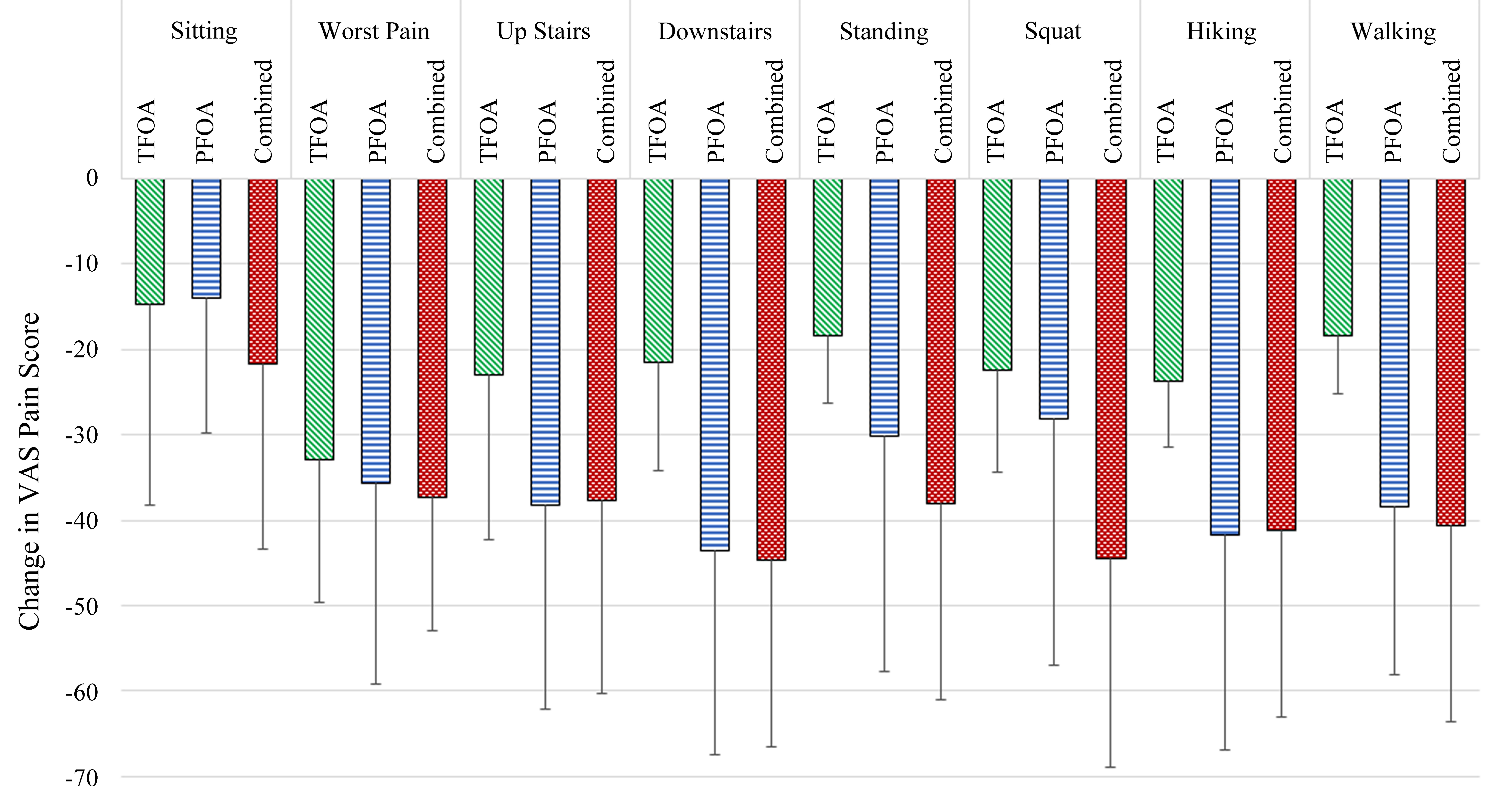


**Supplemental Figure 1.** Change in VAS pain scores (average + SD) from before to after using the TCU brace for specified activities of daily living by symptom group.
