# Supplementary material for: Preliminary Evaluation of a New Orthotic for Patellofemoral and Multicompartment Knee Osteoarthritis
Source: Rehabil Res Pract. 2021 Sep 6;2021:5923721. doi: 10.1155/2021/5923721 (PMC8441257; doi:10.1155/2021/5923721)
Supplement: Supplementary 2 — Appendix 1: retrospective survey. [file 5923721.f2.docx]

**Appendix 1: Retrospective Survey**

**Demographic Information:**

How old are you: _______ years.

What is your current weight: ________ lbs.

What is your height: ____ ft ____ in.

**Knee Pain:**

Which best describes your knee pain **without use of the brace**.

- Pain under the knee cap or front of the knee that typically worsens with walking on inclined terrain, going up and down stairs, squatting or rising from a seated position.
- Pain in the center of the knee that typically worsens with standing for a long period or walking on a flat surface.
- Both of the above.

Which best describes your degree of knee pain **without use of the brace**.

- Pain after a long day of walking or running, greater stiffness in the joint when it’s not used for several hours, or tenderness when kneeling or bending.
- Frequent pain when walking, running, bending, or kneeling. Possible joint stiffness after sitting for long periods of time or when waking up in the morning. Possible joint swelling after extended periods of motion.
- Great pain and discomfort when walking or moving the joint. Knee pain may limit ability to perform activities such as walking, climbing or descending stairs or squatting.

To help people say how much pain they feel, we have drawn a scale (rather like a thermometer) on which no pain is marked 0 and the worst pain you can imagine is marked 100.

In the next three questions, we would like you to indicate on this scale the amount of pain you experience (or experienced) in your knee, in your opinion.

Please do this by marking whichever point on the scale indicates the amount of pain you experience or experienced, as applicable to the question.

| What is the worst pain you experienced at least weekly ***before*** you received the Levitation knee brace? | What is the worst pain you experience at least weekly ***today with use*** the Levitation knee brace? |
| --- | --- |
| 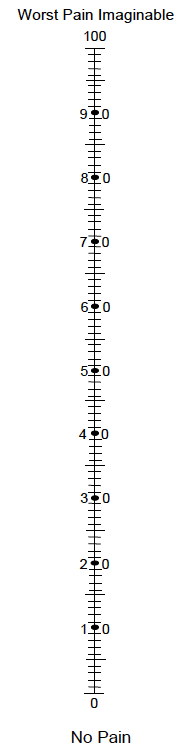 | 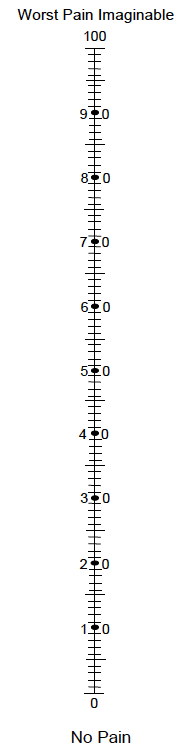 |

In the next questions, we would like you to indicate on the scale the amount of pain you experience (or experienced) in your knee, **during specific activities.**

| **Before receiving the Levitation brace**, what was the amount of pain you typically experienced doing each of the following: | | | | | |
| --- | --- | --- | --- | --- | --- |
| When sitting for a while | Going up or down stairs (whichever worse) | Standing from seated | Crouching or Squatting | Hiking or walking on uneven terrain | Walking long distances on a flat surface |
| 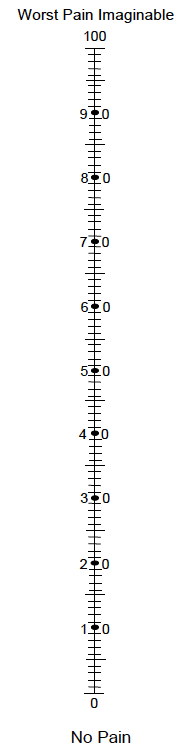 | 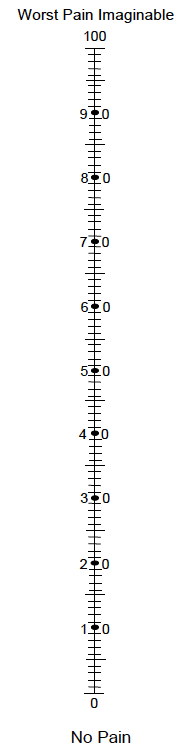 | 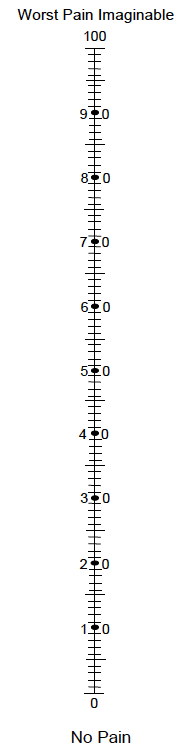 | 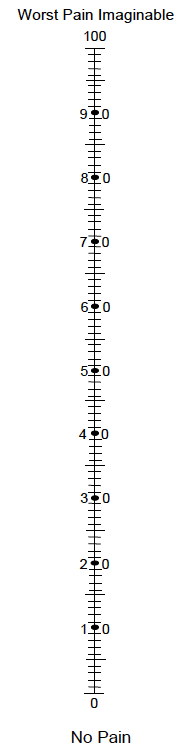 | 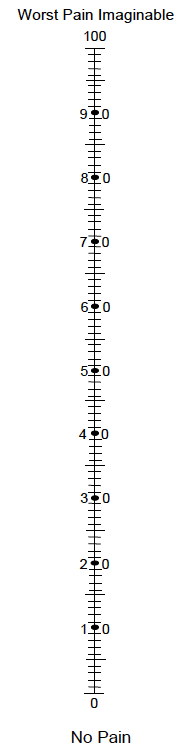 | 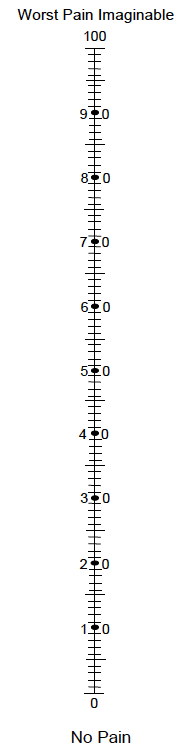 |

| **With the Levitation brace**, what was the amount of pain you typically experience doing each of the following: | | | | | |
| --- | --- | --- | --- | --- | --- |
| When sitting for a while | Going up or down stairs (whichever worse) | Standing from seated | Crouching or Squatting | Hiking or walking on uneven terrain | Walking long distances on a flat surface |
| 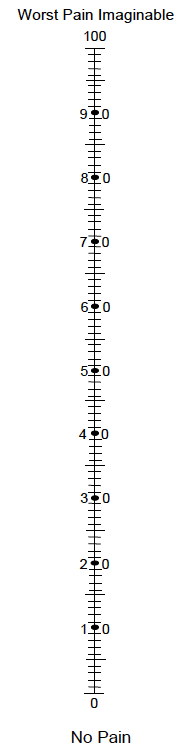 | 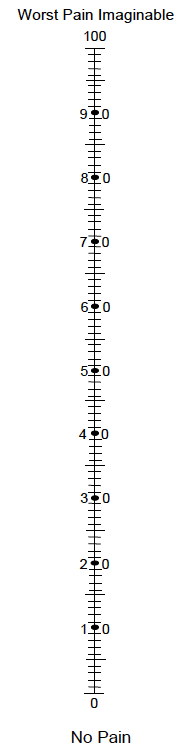 | 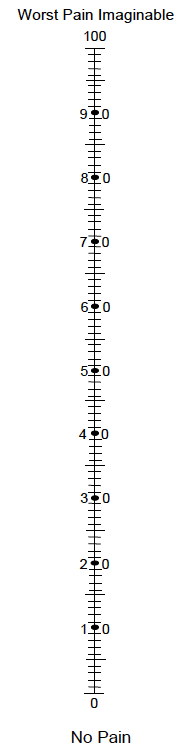 | 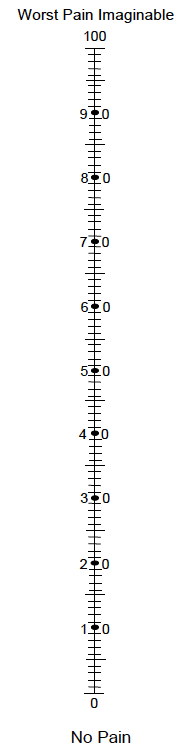 | 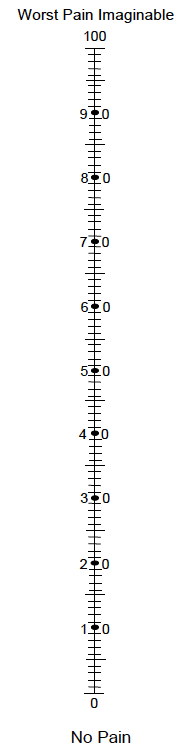 | 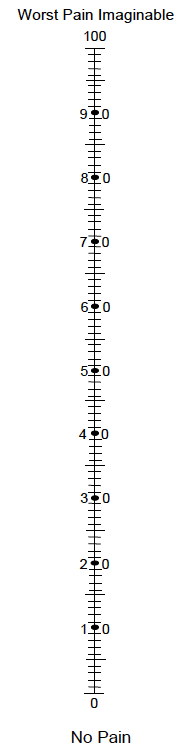 |

**Physical Activity:**

On average, how many hours per week were you physically active for the 3 months ***prior* to using Levitation**?

_________ hours.

On average, how many hrs per week are you physically active **now**?

_________ hours.

**Medication Use:**

List all prescription, over the counter, or other drugs you have used to reduce your knee pain or inflammation *before* and *after* you started using Levitation.

| Name of Medication | Typical dosage before you started using Levitation | Typical dosage now you started using Levitation |
| --- | --- | --- |
| Example: Aspirin | *Example:* 300 mg per day | *Example:* 300 mg per day |
|  |  |  |
|  |  |  |
|  |  |  |
|  |  |  |
|  |  |  |
|  |  |  |
|  |  |  |

**Use of Other Therapies:**

For the 6 months *prior* to using Levitation, did you *use* or *plan* on using any of the following treatment strategies to manage pain in your knee? Select all that apply.

**Injections:**

- Cortisone/Corticosteroid injections
- Hyaluronic acid injections
- Platelet-rich plasma injections

**Allied Health Services:**

- Physiotherapy
- Osteopathy
- Acupuncture
- Occupational therapy
- Chiropractic
- Massage

**Minor Surgery:**

- Arthrocentesis (joint fluid aspiration)
- Arthroscopic surgery

**Major Surgery:**

- Knee replacement surgery

**Recreational Drugs:**

- Medical Marijuana
- Recreational drugs
- Alcohol

**Other Aids:**

- Orthotics
- Other___________________________________

Has Levitation allowed you to reduce, delay, or eliminate your need for any of the following treatments? Select all that apply.

**Injections:**

- Cortisone/Corticosteroid injections
- Hyaluronic acid injections
- Platelet-rich plasma injections

**Allied Health Services:**

- Physiotherapy
- Osteopathy
- Acupuncture
- Occupational therapy
- Chiropractic
- Massage

**Minor Surgery:**

- Arthrocentesis (joint fluid aspiration)
- Arthroscopic surgery

**Major Surgery:**

- Knee replacement surgery

**Recreational Drugs:**

- Medical Marijuana
- Recreational drugs
- Alcohol

**Other Aids:**

- Orthotics
- Other___________________________________

**Lower Extremity Function:**

In the next series of questions, we are interested in knowing whether you are having any difficulty at all with the activities listed because of your knee(s), with and without Levitation. Please provide an answer for **each** activity.

**Prior to using the Levitation brace** did you or would you have any difficulty at all with:

| Activities | Extreme Difficulty or Unable to Perform Activity | Quite a Bit of Difficulty | Moderate Difficulty | A Little Bit of Difficulty | No Difficulty |
| --- | --- | --- | --- | --- | --- |
| Any of your usual work, housework, or school activities. | 0 | 1 | 2 | 3 | 4 |
| Your usual hobbies, recreational or sporting activities. | 0 | 1 | 2 | 3 | 4 |
| Getting into or out of the bath. | 0 | 1 | 2 | 3 | 4 |
| Walking between rooms. | 0 | 1 | 2 | 3 | 4 |
| Putting on your shoes or socks. | 0 | 1 | 2 | 3 | 4 |
| Squatting. | 0 | 1 | 2 | 3 | 4 |
| Lifting an object, like a bag of groceries from the floor. | 0 | 1 | 2 | 3 | 4 |
| Performing lightweight activities around your home. | 0 | 1 | 2 | 3 | 4 |
| Getting into or out of a car. | 0 | 1 | 2 | 3 | 4 |
| Walking 2 blocks. | 0 | 1 | 2 | 3 | 4 |
| Walking a mile. | 0 | 1 | 2 | 3 | 4 |
| Going up or down 10 stairs (about 1 flight of stairs). | 0 | 1 | 2 | 3 | 4 |
| Standing for 1 hour. | 0 | 1 | 2 | 3 | 4 |
| Sitting for 1 hour. | 0 | 1 | 2 | 3 | 4 |
| Running on even ground. | 0 | 1 | 2 | 3 | 4 |
| Making sharp turns while running fast. | 0 | 1 | 2 | 3 | 4 |
| Hopping. | 0 | 1 | 2 | 3 | 4 |
| Rolling over in bed. | 0 | 1 | 2 | 3 | 4 |

**With use of the Levitation knee brace today** do you or would you have any difficulty at all with:

| Activities | Extreme Difficulty or Unable to Perform Activity | Quite a Bit of Difficulty | Moderate Difficulty | A Little Bit of Difficulty | No Difficulty |
| --- | --- | --- | --- | --- | --- |
| Any of your usual work, housework, or school activities. | 0 | 1 | 2 | 3 | 4 |
| Your usual hobbies, recreational or sporting activities. | 0 | 1 | 2 | 3 | 4 |
| Getting into or out of the bath. | 0 | 1 | 2 | 3 | 4 |
| Walking between rooms. | 0 | 1 | 2 | 3 | 4 |
| Putting on your shoes or socks. | 0 | 1 | 2 | 3 | 4 |
| Squatting. | 0 | 1 | 2 | 3 | 4 |
| Lifting an object, like a bag of groceries from the floor. | 0 | 1 | 2 | 3 | 4 |
| Performing lightweight activities around your home. | 0 | 1 | 2 | 3 | 4 |
| Getting into or out of a car. | 0 | 1 | 2 | 3 | 4 |
| Walking 2 blocks. | 0 | 1 | 2 | 3 | 4 |
| Walking a mile. | 0 | 1 | 2 | 3 | 4 |
| Going up or down 10 stairs (about 1 flight of stairs). | 0 | 1 | 2 | 3 | 4 |
| Standing for 1 hour. | 0 | 1 | 2 | 3 | 4 |
| Sitting for 1 hour. | 0 | 1 | 2 | 3 | 4 |
| Running on even ground. | 0 | 1 | 2 | 3 | 4 |
| Making sharp turns while running fast. | 0 | 1 | 2 | 3 | 4 |
| Hopping. | 0 | 1 | 2 | 3 | 4 |
| Rolling over in bed. | 0 | 1 | 2 | 3 | 4 |
